# Four and A-Half Inches of Whalebone in the Uterus

**Published:** 1868-04

**Authors:** M. M. Eaton

**Affiliations:** Peoria, Ill.


ARTICLE XIII.

### FOUR AND A-HALF INCHES OF WHALEBONE IN THE UTERUS.—ABORTION.—NARROW ESCAPE FROM DEATH.

By M. M. EATON, M.D., Peoria, Ill.

March 30th, 1866, was called, in consultation, to see Mrs. G., living in Tazewell Co. Met the attending physician at her residence, who gave me the history of the case, as follows, *viz.:* He was called to see Mrs. G., aged about 30 years, March 29th; found her flowing profusely. She stated that she had had a miscarriage three days before, the afterbirth also being all delivered. He made an examination, but found nothing abnormal, save that the uterus was enlarged, the os somewhat dilated, and a general relaxed condition. He made use of the usual remedies and appliances. March 30th, was called to see Mrs. G. in great haste, the messenger stating that she was very low. On arriving at the house, a woman in attendance stated that Mrs. G. had told her that she had used a piece of whalebone to bring on a miscarriage, and had lost it in the womb. The Doctor found his patient in a truly alarming condition—almost speechless and pulseless, with the bed saturated with blood, and still flowing. On making a vaginal examination, and reaching very high up into the uterus, he detected some hard substance, but was unable to remove it; when he requested that I be sent for.

On entering the lady's chamber, I found her colorless, nearly pulseless, and hardly able to speak a word. Ordered her to have a drink of warm water with rye whiskey, and cold applied over the hypogastric region. In about 15 minutes, the pulse became stronger, and I proceeded, by request of the Doctor, to attempt the removal of the whalebone, if such it was. I easily reached the object with one finger, but the os was not large enough to admit of forceps being used at the same time, and I was unable to sieze the whalebone with them. The whalebone lay transversely in the uterus, and I made an effort to bring down one end with my finger, and finally succeeded in hooking my finger over it and bringing it down; but it doubled on itself, and I removed it forcibly, without letting go my hold. I found the substance to be a very large whalebone (such as is used in umbrella frames), 4½ inches in length, slightly pointed at both extremities. Ergota was immediately given, and followed by stimulants and tonics. I have since learned from the attending physician that she ultimately recovered.

I wish every woman in the land who is, or is tempted to be, guilty of the crime of producing abortion on themselves, or anyone else, could have seen this case. I am sure, if they could have done so; few would be found willing to subject themselves to such danger. This is but one out of thousands, it is true, where women resort to some means to rid themselves of unborn children; but, it is one calculated to impress the thoughtful mind with the danger women subject themselves to, who sin in this way against God and man, and furnishes us with a case to cite, to persuade ladies never to be guilty of such an act.

What numbers of American women are, to-day, dragging out a miserable existence themselves, and giving birth to scrawny, weakly children, because of their having taken so many drugs, "to get rid of them," as they say. I am sure many are barren who would now gladly have children if they could; but they cannot, because of the fact that they, when first married, thought to be smart, and not have any children until they got ready, and the means used either to prevent or destroy conceptions, has resulted in a permanent disability to conceive. Many are troubled with ulcerations, hemorrhages, tumors, misplacements, and inflammations, I conjecture, nay, I have been informed by the parties themselves in many instances, as they believe (and I judge, correctly), by reason of their efforts to rid themselves of the trouble of raising children; but a greater trouble has been visited upon them, and they uniformly repent their acts. Let others take warning.

